# Longitudinal Associations Between Biopsychosocial Factors and Sustainable Return to Work of Sick-Listed Workers with a Depressive or Anxiety Disorder

**DOI:** 10.1007/s10926-015-9588-z

**Published:** 2015-06-21

**Authors:** Lieke Lammerts, Frederieke G. Schaafsma, Merijn Eikelenboom, Sylvia J. Vermeulen, Willem van Mechelen, Johannes R. Anema, Brenda W. J. H. Penninx

**Affiliations:** Department of Public and Occupational Health, EMGO+ Institute for Health and Care Research, VU University Medical Center, P.O. Box 7057, 1007 MB Amsterdam, The Netherlands; Research Centre for Insurance Medicine, AMC-UMCG-UWV-VUmc, Amsterdam, The Netherlands; Department of Psychiatry, EMGO+ Institute for Health and Care Research, VU University Medical Center/GGZ inGeest, Amsterdam, The Netherlands; Department of Psychiatry, Leiden University Medical Center, Leiden, The Netherlands; Department of Psychiatry, University Medical Center Groningen, University of Groningen, Groningen, The Netherlands

**Keywords:** Long-term sickness absence, Mental health problems, Return to work

## Abstract

*Purpose* Only a limited number of studies have investigated return to work of sick-listed workers with mental health problems, and more knowledge is needed about the influence of non-disorder-related factors. This study aimed to identify longitudinal associations between demographic, personality, disorder-related and work-related characteristics and sustainable return to work of sick-listed workers with a depressive or anxiety disorder. *Methods* We used data of a large Dutch cohort study to prospectively study longitudinal associations between biopsychosocial factors and sustainable return to work in 2 years. Associations were studied by means of univariable and multivariable logistic regression analysis. Participants who were sick-listed at baseline and had a lifetime diagnosis of a depressive and/or anxiety disorder were included in this study (N = 215). *Results* In 2 years, 51.6 % of the participants returned to work sustainably. Age, household income, extraversion, employment status, skill discretion and job security were significantly (*P* ≤ 0.05) associated with sustainable RTW in 2 years in the univariable analyses. The multivariable analysis revealed significant associations between sustainable return to work and age (OR per 10 years = 0.67; 95 % CI 0.47–0.95), household income (OR per 100 Euro’s a month = 1.04; 95 % CI 1.00–1.08) and being on sickness benefit versus being (self-)employed (OR 0.39; 95 % CI 0.20–0.77). *Conclusions* In the long-run not disorder-related factors, but an older age, the absence of a job and a low household income seem to complicate return to work. Policy and research should focus on facilitators and barriers for return to work of workers with these characteristics.

## Introduction

As a result of high rates of long-term sickness absence many countries since the 1990s have aimed to improve the return to work (RTW) of sick-listed workers [1]. Mental health problems have been a major cause of these high (long-term) sickness absence rates. In 2012 the OECD reported an increase in the proportion of disability benefits that was granted on the grounds of a mental disorder from 15 to 25 % in the mid-1990s to 30–50 % in 2009/10 [2]. In addition, numerous studies have identified the presence or symptoms of mental health problems, like depression or distress, as important risk factors for long-term sickness absence [3–7]. These high rates of long-term sickness absence caused by mental health problems have been an important public health concern, as it affects both the individual and society as a whole [8]. Loss of independence, uncertainty, changed self-perception and changed economic conditions have been reported by sick-listed workers in a qualitative study [9]. For society, mental health problems and related sickness absence often result in high costs. To illustrate, in the US in the late 1990s the economic burden of depression and other mental health problems was already one of the highest in comparison with the burden of other illnesses [10].

Policies aiming to improve RTW of (long-term) sick-listed workers include incentives for employers and employees towards reintegration of sick-listed workers, an increase in employment programs, vocational rehabilitation and stricter requirements for approval of disability claims [1]. Characteristics of sick-listed workers have often been examined in previous research [3–6, 11–14]. In order to make policies for RTW succeed, it is not only important to know which workers are more prone for long-term sickness absence, but it is also relevant to consider which characteristics of these sick-listed workers affect their RTW. In his editorial on long term sickness absence, Henderson [8] states that ‘longer absences are associated with a reduced probability of eventual RTW’. In order to prevent long absences and to facilitate sustainable RTW, policy makers should be aware of factors that have a long-term influence on the (sustainability of) RTW of (long-term) sick-listed workers.

From occupational health practice we know that RTW of sick-listed workers is dependent on several factors, e.g. perceived health status, employment history and age of the sick-listed worker [15]. Different theories, such as the biopsychosocial model, also suggest that the ability to work actually results from a combination of biological, psychological and social factors [16, 17]. Systematic reviews of the literature have revealed that only a limited number of studies have investigated factors associated with RTW of sick-listed workers with mental health problems [11, 18, 19] and more knowledge is needed about the influence of other types of factors than the ones that are disorder-related, such as work-related and personal factors [18, 19]. Vlasveld and colleagues [14] found associations between long-term sickness absence and several personality traits, i.e. high neuroticism, external locus of control, low extraversion and low conscientiousness. They recommended further research on the influence of personality traits on RTW. The object of our prospective study was to take all these factors into account and to identify longitudinal associations between a broad range of factors and sustainable RTW in 2 years of sick-listed workers with a depressive or anxiety disorder, two common mental disorders [20]. In this study we addressed demographic, personality, disorder-related and work-related characteristics.

## Methods

### Design and Procedure

In order to identify factors that are associated with sustainable RTW of sick-listed workers with a depressive or anxiety disorder, data of NESDA (‘The Netherlands Study of Depression and Anxiety’) was used. NESDA is a Dutch longitudinal multi-site naturalistic cohort study. The aim of NESDA is to study the long-term course of depressive and anxiety disorders among 2981 participants aged 18–65 years. NESDA provides detailed information about the severity, type and duration of the disorder and contains a careful documentation of the participants’ work status and current or last profession, the participants’ personality traits and demographic characteristics.

At the onset of NESDA, 1701 participants had been shortly before diagnosed with a depressive and/or anxiety disorder. At that point 907 participants had a life-time diagnosis, which means that they had had a depressive or anxiety disorder at least once in their lives, or an increased likelihood to develop a depressive or anxiety disorder, because of their family history or because of sub-threshold depressive or anxiety symptoms. The remaining 373 participants were healthy controls. Participants were recruited from community samples (which were the NEMESIS [21] and the ARIADNE cohorts [22]), through mental health care organizations (when newly enrolled at one of the 17 participating centers) and through primary care practices (by using a three-stage screening procedure). Only two exclusion criteria were used: (1) a primary clinical diagnosis of a psychiatric disorder not subject of NESDA and (2) not being fluent in Dutch. The NESDA study protocol was approved by the Ethical Review Board of participating institutes and all respondents signed a written informed consent. The rationale, objectives and methods of NESDA are described in detail elsewhere [23]. For this study we used baseline data of NESDA (T0), data of the first face-to-face follow-up measurement 2 years after the baseline measurement (T1), and data of the second face-to-face follow-up measurement 4 years after the baseline measurement (T2).

In our analyses we included all participants of NESDA in our analysis who had a lifetime diagnosis of a depressive or anxiety disorder at T0 and who were sick-listed at T0 or T1. For participants who were included on the basis of their sickness absence during T1, the data collected during this measurement moment was considered as baseline data. In case data were missing at T1 but available at T0, these data were used to determine the baseline characteristics of this group. The CIDI (WHO version 2.0) was used by specially trained clinical research staff to determine diagnoses of depressive and anxiety disorders according to the DSM-IV criteria [24]. Employment status and sickness absence were assessed with the Trimbos/iMTA questionnaire for costs associated with Psychiatric illness (Tic-P) [25]. Participants had either indicated that they were sick-listed from a paid job for more than 6 months or that they received sickness benefit. The latter group was included irrespectively of the duration of their benefit. Participants who were more than 80 % occupationally disabled at baseline were excluded, since, according to the Dutch law, these participants can be considered being sustainably occupationally disabled. Other exclusion criteria were: (1) being (early) retired at baseline; (2) being on pregnancy/maternity leave at baseline and/or during the follow-up measurement; (3) no participation in the follow-up measurement and (4) having been sick-listed for <14 days in the previous 6 months at baseline. With this threshold of 2 weeks we differentiated between absenteeism of <2 weeks, most likely related to a cold or the flue, and longer absenteeism that may be caused by a chronic condition [26]. As a result, 215 participants were included in our study: 176 participants at T0 and 39 participants at T1.

### Measures

#### Dependent Variable

The primary outcome measure was sustainable RTW in 2 years. Sustainable RTW was operationalized as follows: the participant is (self-)employed and has not been long-term sick-listed (more than 14 days) in the previous 6 months. Data collected with the Tic-P [25], during T1 and T2 of NESDA, were used to assess the primary outcome.

#### Independent Variables

The selection of independent variables was based on the biopsychosocial model. According to this model, work participation or disability of people with health problems includes a biological, psychological and social dimension [17]. The biological dimension normally refers to the health condition. As there are (often) no biomarkers that indicate the presence or symptoms of mental disorders, work participation of sick-listed workers with mental disorders has no clear biological dimension. However, also mental disorders result in ill health and characteristics of these disorders should be taken into account. The psychological dimension of the biopsychosocial model recognizes the influence of personal factors and the social dimension consists of the social context, pressures and constraints, including characteristics of the working environment [17]. Based on these dimensions, a distinction was made in demographic, personality, disorder-related and work-related characteristics of the sick-listed worker.

##### Demographic Characteristics

The following self-reported demographic characteristics were taken into account: (a) gender; (b) age (in years); (c) education (in years); (d) marital/partner status (partner vs. no partner); and (e) net income of the household in Euros per month.

##### Personality Characteristics

The personality characteristics that were included were: (a) neuroticism; (b) extraversion; (c) openness; (d) agreeableness; (e) conscientiousness; and (f) locus of control. Neuroticism, extraversion, openness, agreeableness and conscientiousness together form The Big five personality characteristics. In NESDA the NEO-FFI questionnaire was used to measure these five domains of personality. This questionnaire consists of 12 items per domain, measured on a five-point Likert response format [27]. Locus of control was assessed by a translated five-item abbreviated version of the Pearlin Mastery Scale [28], with a range from 5 to 25. Higher scores on this scale indicate more feelings of mastery.

##### Disorder-Related Characteristics

The following disorder-related characteristics were assessed: (a) diagnoses of depressive or anxiety disorders (no current depressive or anxiety disorder; current depressive disorder; current anxiety disorder; comorbidity between a current depressive and anxiety disorder); (b) severity of depressive symptoms; (c) severity of anxiety symptoms; (d) duration of depressive symptoms; (e) duration of anxiety symptoms; (f) use of antidepressants (frequent use versus no or infrequent use); and (g) treatment by specialized mental health care professionals in the preceding 6 months (specialized mental health care vs. no specialized mental health care).

In NESDA the CIDI was used to assess the diagnosis of a depressive or anxiety disorder according to the DSM-IV criteria [24]. If a disorder could have been diagnosed within the preceding 6 months, this was labeled as a current disorder. Severity of depressive symptoms was assessed with the 28-item inventory of depressive symptomatology self-report version [29]. Each item of this questionnaire contains four answer categories that correspond to a score ranging from zero to three. The 21-item Beck Anxiety Inventory [30] was used to measure severity of generalized anxiety and panic symptoms. This questionnaire also uses a four-point scale ranging from zero to three. The duration of depressive and anxiety symptoms was measured with the life chart interview [31]. Using a calendar event recall method, the participant was asked about the course of complaints. The recall period was 5 years for participants included at T0 and 2 years for participants included at T1. Based on the description of the course of complaints, a measure for the duration of symptoms was constructed. This measure was expressed in percentage of time. During the face-to-face measurements in NESDA also the use of antidepressants was quantified. Use of the medicine for more than 50 % of the days in the preceding 6 months was coded as frequent use. Besides the use of antidepressants, also more specialized mental health care was taken into account. With the use of the Tic-P [25] the number of visits to different specialized mental health care professionals was quantified. We differentiated between participants who had more than one contact with a first line psychologist, a social worker, a social psychiatric nurse, an institute for mental health care, an independent psychiatrist or a psychotherapist in the preceding 6 months and participants who had not.

##### Work-Related Characteristics

Based on the information about the employment status of the participants at baseline, it was possible to differentiate between participants who had indicated that they were self-employed, participants who had an employment contract, participants who had indicated that they were partly occupationally disabled and participants who were on sickness benefit. In the Netherlands, people who become sick-listed and who have no (longer an) employment contract can apply for a sickness benefit at the Dutch social security agency (SSA). We decided to make a distinction between sick-listed workers who were still employed and sick-listed workers who were on sickness benefit or partly occupationally disabled and therefore had a more vulnerable position on the labor market [32].

In NESDA the Job Content Questionnaire (JCQ) [33] was used at baseline to assess conditions in the current or last workplace. The JCQ consists of five subscales, with a sum score per subscale ranging from zero to one: job demands, decision authority, skill discretion, social support at work and job security. The sum scores of the sub scales were dichotomized based on the median split. As previously done by Holleman and colleagues [34], the median split of job demands and decision authority was used to create a new variable, i.e. job strain, which distinguishes people with high job demands and low decision authority from others. In previous research of NESDA the type of current or last profession was constructed by using an occupational code provided by Statistics Netherlands (CBS) and additional self-reported information on employment status and supervisory status assessed with the use of the JCQ [35]. We used this classification to differentiate between blue and white-collar workers.

As a result, the following work-related variables were taken into account: (a) employment status (vulnerable worker vs. being (self-)employed); (b) duration of sickness absence (longer vs. shorter than 6 months); (c) skill discretion (high vs. low); (d) social support at work (high vs. low); (e) job security (high vs. low); (f) job strain (job strain vs. no job strain); and (g) type of current or last profession (blue vs. white collar).

### Analysis

#### Missing Value Analysis

*T* tests with groups formed by indicator variables and cross tabulations of categorical and indicator variables were performed to investigate if the pattern of missing data in one variable affected the values of another variable. In addition, the hypothesis that the data were missing completely at random was tested with the Little’s MCAR test.

#### Analysis of Associations

Descriptive analyses were used to describe the study population at baseline. Logistic regression analysis was used to determine which factors were associated with RTW in 2 years. Univariable logistic regression analysis was performed for all independent variables, with sustainable RTW in 2 years as the dependent variable. Variables that had a *P* value <0.15 in the univariable analysis were entered into a combined multivariable logistic regression model. A cut-off value of *P* ≤ 0.05 was used to determine the significance of the associations in the combined model (Wald statistic). Multicollinearity between the variables in the combined model was checked by means of multicollinearity diagnostics. When the resulting VIF scores were >10, multicollinearity was assumed [36]. In addition, correlations between variables were investigated if these variables were likely to measure the same construct. SPSS version 20.0 was used for the statistical analysis.

## Results

### Characteristics of the Study Population at Baseline

Characteristics of the study population at baseline are summarized in Table 1. More than 90 % of all participants were currently diagnosed with a depressive or anxiety disorder at baseline, of which slightly more than half had a combination of a current depressive and anxiety disorder. About three-fourths of all participants was at baseline sick-listed for more than 6 months. More than half of the participants, 62.3 %, could be labeled as a vulnerable sick-listed worker. Most of them, about 98 %, had indicated that they were on sickness benefit.

**Table 1:** Characteristics of the study population^a^

Baseline characteristics	
*Demographic characteristics*	
Sex, % female	66.5
Age (range 20–62), mean (SD)	42.32 (10.53)
Partner status, % partner or married	67.9
Education in years (range 5–18)	11.74 (3.29)
Net income of household in Euros a month	2244.86 (1020.42)
(range <600–>5000), mean (SD)	
*Personality characteristics*	
Neuroticism (range 18–57), mean (SD)	41.16 (7.40)
Extraversion (range 15–52), mean (SD)	33.95 (6.88)
Openness (range 24–57), mean (SD)	37.71 (5.90)
Agreeableness (range 28–59), mean (SD)	43.74 (5.20)
Conscientiousness (range 19–57), mean (SD)	40.18 (7.27)
Locus of control (range 5–25), mean (SD)	14.51 (4.20)
*Disorder*-*related characteristics*	
Diagnosis anxiety or depression	
No current depressive or anxiety disorder (%)	8.4
Current depressive disorder (%)	21.4
Current anxiety disorder (%)	16.7
Comorbidity between depressive and anxiety disorder (%)	53.5
Severity depression (range 3–58), mean (SD)	32.02 (12.91)
Severity anxiety (range 0–58), mean (SD)	18.41 (11.06)
Percentage of time depressive symptoms (range 0–100), mean (SD)	33.70 (30.83)
Percentage of time anxiety symptoms (range 0–100), mean (SD)	40.52 (35.28)
Use of antidepressants, % frequent use	52.1
Specialized mental health care, used by %	76.7
*Work*-*related characteristics*	
Employment status, % vulnerable worker	62.3
Sickness absence, % more than 6 months	73.0
Job demands (range 0–1), mean (SD)	0.54 (0.37)
Decision authority (range 0–1), mean (SD)	0.66 (0.33)
Skill discretion (range 0–1), mean (SD)	0.68 (0.30)
Social support (range 0–1), mean (SD)	0.58 (0.32)
Job security (range 0–1), mean (SD)	0.54 (0.41)
Type of worker	
White collar (%)	77.8
Blue collar (%)	22.2

^a^N varies between 171 and 215 due to missing cases

Data about the personality traits, assessed with the NEO-FFI questionnaire and the Pearlin Mastery Scale, were missing for two to ten percent of the participants. Data about the work-related characteristics that were measured with the JCQ (skill discretion, social support at work, job security, job strain and type of current or last profession) were missing for 17–33 % of the participants. The missing value analysis showed that there was no significant difference between the participants with and without missing values (Little’s MCAR test, *P* = *0.186*).

### Associations with Sustainable RTW in Two Years

In 2 years, 51.6 % of the participants returned to work sustainably. All associations with sustainable RTW in 2 years, both univariable and multivariable, are summarized in Table 2.

**Table 2:** Univariable and multivariable associations with sustainable RTW in 2 years^a^

Baseline characteristics^b^	Univariable associations^c^			Multivariable associations in combined model^c^		
	OR	95 % CI	*P*	OR	95 % CI	*P*
*Demographic characteristics*						
Sex, female	0.73	0.41–1.28	0.27			
Age (per 10 years increase)	0.71	0.54–0.92	0.01	0.67	0.47–0.95	0.02
Education (per year increase)	1.08	0.99–1.17	0.08	1.01	0.91–1.13	0.83
Partner status, partner	1.25	0.71–2.22	0.44			
Net income of household (per 100 Euro’s a month increase)	1.04	1.01–1.07	<0.01	1.04	1.00–1.08	0.04
*Personality characteristics* ^d^						
Neuroticism	0.89	0.68–1.17	0.41			
Extraversion	1.33	1.00–1.75	0.05	1.25	0.87–1.78	0.23
Openness	0.92	0.70–1.21	0.54			
Agreeableness	1.01	0.77–1.33	0.92			
Conscientiousness	1.27	0.97–1.68	0.09	1.03	0.71–1.49	0.90
Locus of control	1.03	0.78–1.37	0.82			
*Disorder*-*related characteristics*						
Diagnosis anxiety or depression			0.68			
No current depressive or anxiety disorder	REF	–	–			
Current depressive disorder	0.67	0.23–2.01	0.48			
Current anxiety disorder	0.72	0.23–2.23	0.56			
Comorbidity	0.97	0.36–2.63	0.95			
Severity depression	0.99	0.97–1.01	0.19			
Severity anxiety	1.00	0.98–1.03	0.92			
Duration of depressive symptoms (per 10 % time increase)	0.95	0.87–1.04	0.29			
Duration of anxiety symptoms (per 10 % time increase)	0.98	0.91–1.06	0.68			
Frequent use of antidepressants	1.37	0.80–2.34	0.25			
Specialized mental health care	1.09	0.58–2.05	0.79			
*Work*-*related characteristics*						
Employment status, vulnerable worker	0.37	0.21–0.66	<0.01	0.39	0.20–0.77	<0.01
Sickness absence >6 months	0.75	0.41–1.37	0.35			
Job strain	0.97	0.52–1.79	0.92			
High skill discretion	1.90	1.05–3.46	0.04	1.47	0.73–2.98	0.28
High social support	1.45	0.79–2.64	0.23			
High job security	2.05	1.11–3.78	0.02	1.44	0.71–2.92	0.31
Type of worker, blue collar	0.71	0.32–1.57	0.40			

^a^N varies between 171 and 215 due to missing cases

^b^The reference category for each dichotomous variable is the contrast (‘female vs. male’)

^c^Reference category is ‘no sustainable RTW in 2 years’

^d^OR’s are per SD increase. SD neuroticism is 7.40; SD extraversion is 6.88; SD openness is 5.90; SD agreeableness is 5.20; SD conscientiousness is 7.27; SD locus of control is 4.20

In the univariable analysis the following baseline characteristics had an association of *P* < 0.15 with sustainable RTW in 2 years and were selected for multivariable analysis: age, education, net income of the household, extraversion, conscientiousness, employment status, skill discretion and job security. None of the disorder-related factors was significantly (*P* ≤ 0.05) associated with RTW in 2 years.

In the combined model significant associations were found between sustainable RTW in 2 years and age, net income of the household and employment status. The OR for sustainable RTW per 10 years age increase was 0.67 (95 % CI 0.47–0.95), indicating lower odds of sustainable RTW at a higher age. This OR was 1.04 (95 % CI 1.00–1.08) per increase of 100 Euros a month in net income of the household, which means that one is more likely to return to work sustainably at a higher household income level. Being a vulnerable worker compared to a (self-)employed worker resulted in a more than two times smaller odds of sustainable RTW (OR 0.39; 95 % CI 0.20–0.77). All the VIF-scores in the collinearity statistics for the combined model were <10.00, so multicollinearity was not assumed.

## Discussion

### Main Findings

The aim of this study was to investigate longitudinal associations between demographic, personality, disorder-related and work-related characteristics and sustainable RTW in 2 years of sick-listed workers with a lifetime diagnosis of a depressive or anxiety disorder. In 2 years, 51.6 % of the study participants returned to work sustainably. This study revealed that in the long-run not disorder-related factors, but a younger age, a higher household income level and being (self-)employed are all together associated with a higher odds of sustainable RTW in 2 years of sick-listed workers with a depressive or anxiety disorder.

### Comparison with Other Studies

Most of the participants in this study had currently been diagnosed with a depressive and/or anxiety disorder at baseline. Earlier research within NESDA reported a twofold and a sevenfold higher risk of long-term sickness absence for persons with respectively an anxiety disorder or depressive disorder in the same period that the disorder was present, so cross-sectional [26]. We selected participants of NESDA for our study, based on their long-term sickness absence. Since participants with a depression had the highest risk of long-term sickness absence, it is not surprising that many of our respondents were diagnosed with a current depression at baseline. Another study within NESDA revealed that persons with a depression, are also most likely to have recovered in 2 years [37]. This might be an explanation for the absence of an association between the presence or severity of the disorder at the moment of sick-listing and RTW 2 years later. Moreover, our findings confirm that when one’s aim is to enhance sustainable RTW of sick-listed workers with mental health problems, it is not sufficient to solely focus on characteristics of the disorder itself, which has often been done in previous studies [18].

The influence of a broad range of factors on RTW has been studied before in study populations consisting of sick-listed workers with physical complaints, such as low back pain. Results of these studies emphasize the importance of work-related factors in RTW, such as job satisfaction, social support, job demands and job control [38–41]. In our study, univariable associations were found between sustainable RTW in 2 years and two work-related factors: a high job security and a high skill discretion. However, in the combined model, the associations between sustainable RTW and these work-related factors, did not remain significant. This may be explained by the high number of participants that was on sickness benefit at baseline. They probably had no (longer a) workplace to return to, so that characteristics of the job influenced RTW to a lesser extent.

More than half of the participants in our study reported at baseline that they were on sickness benefit. They had a more than two times lower odds of returning to work in 2 years than participants who at baseline reported that they were (self-)employed. In the Netherlands, unemployed workers, temporary agency workers and workers with an expired fixed-term contract who become sick-listed can apply for a sickness benefit from the Dutch SSA. Both unemployment and temporary employment have been related to poor (mental) health [11, 15, 42–44]. Nevertheless, it seems that these workers are not sick-listed more often [45, 46], but when they do get sick-listed, the absence of a workplace to return to will complicate their RTW importantly [15]. This stresses the need for vocational interventions that create a RTW perspective [47, 48], i.e. interventions that focus on a suitable job for vocational rehabilitation. As evidence for effective vocational interventions for this vulnerable group of workers is lacking, more research on this topic should be promoted.

Besides the absence of a job to return to, also other obstacles for RTW might explain the reduced odds of sustainable RTW in 2 years for sick-listed workers on sickness benefit. It is possible that these workers experience a so called ‘benefit trap’. This means that the perceived (economic) benefits of staying out of work exceed the benefits of returning to work, for example because it is not possible to find a job that pays more than the income from being unemployed or sick-listed [49]. This could also be an explanation for the reduced odds of sustainable RTW in case of a lower household income that was found in this study. A benefit trap might be experienced by the ones with a lower income.

Apart from the sick-listed workers without a (permanent) employment contract, also the older workers seem to represent a vulnerable group. This study showed that the odds of sustainable RTW of sick-listed workers with a depressive or anxiety disorder decreases significantly per each 10 years of age increase. This finding is highly supported by earlier research [7, 11, 18, 19]. As the workforce is ageing, work participation of older workers is of growing importance. Based on an in-depth study of older workers’ perspectives and previous research, Koolhaas and colleagues [50] proposed a tailor-made intervention with the aim to enhance sustainable working life, with a central focus on work-related problems and obstacles, personal development opportunities and environmental factors. Knowledge about the effectiveness of these kinds of interventions for older workers is needed.

### Strengths and Limitations

Systematic reviews of the literature have shown that previous prognostic cohort studies more often addressed disorder-related factors, compared to work-related and personal factors, when studying RTW of sick-listed workers with mental health problems [18, 19]. To our knowledge this has been one of the first studies that paid equal attention to the long-term influence of demographic, personality, disorder-related and work-related characteristics. This made it possible to study the independent effects of all these different factors and this is an important strength of our study.

A second strength of this study is that longitudinal associations were studied. All independent variables were measured at baseline. At this point all participants were sick-listed. In this way, all independent variables were measured prior to the possible occurrence of the outcome. Longitudinal associations provide more information than associations that are determined in a cross-sectional study, because with only cross-sectional data it is not possible to know whether an independent variable preceded the outcome or not. Moreover, assessing longitudinal associations between RTW and multiple factors, makes it possible to determine which of these factors have a long-term influence on RTW. This provides important information for policymakers who are engaged in the development of RTW policies.

Another strength of the study is that participants with a variety in duration of sickness absence and employment status were included in the study, which made it possible to investigate the influence of sickness absence duration and employment status on sustainable RTW. A disadvantage of our selection of participants is that the study population consists of participants with a probably worse prognosis than the source population of NESDA. Therefore, generalizing these results to other groups, such as workers who are only short-term sick-listed from a paid job, may be limited.

Another limitation of the study was the interpretation of the employment status of participants. In NESDA the Tic-P was used to collect information about the employment status of participants. In this study we assumed that the participants who indicated that they were on sickness benefit had no workplace to return to. In the Netherlands being on sickness benefit usually means that someone has applied for a sickness benefit from the Dutch SSA, because of the absence of an employer. However, as employment status was self-reported by the participants, we are not sure if the participants who had indicated that they were on sickness benefit actually had no (longer an) employment contract. Nevertheless, the sick-listed workers who had indicated that they were on sickness benefit differed significantly in outcome from the sick-listed workers who had indicated that they were (self-)employed.

The outcome measure, sustainable RTW in 2 years, was also assessed with the Tic-P [25]. This questionnaire uses a reference period of 6 months. For that reason, it was only possible to know whether the participant had returned to work for a limited period of time (6 months). This is a limitation of our study. However, the follow-up period was more than these 6 months. Our outcome measure was assessed after 2 years follow-up, with a recall period of 6 months. As we were interested in return to work on the long run, the assessment of RTW after 2 years provided us with very valuable information. The measurement of the outcome with the use of the Tic-P, did not only show whether someone was at work in 2 years, but also provided some information about the sustainability of this outcome, because information was available about days of sickness absence in the previous 6 months.

The varying number of participants in the analysis due to missing values is also a limitation. However, the hypothesis that the values were missing completely at random could not be rejected. Imputation of missing data would probably not have provided new information. For that reason, we decided not to apply any data imputation techniques.

### Practical Implications and Further Research

As long-term sickness absence is more and more caused by mental health problems [2], it is for policymakers and occupational health care professionals important to know which (modifiable) factors influence sustainable RTW of sick-listed workers with mental health problems and to anticipate on this. This study reveals that in the long run characteristics of the disorder itself, such as duration and severity, do not influence sustainable RTW. Although work participation of sick-listed workers with mental health problems has still been studied mainly in regard with the disorder itself, there is a growing awareness of the importance of a healthy and steady job. The results of this study indicate that some workers are more vulnerable than others when becoming sick-listed. Especially older workers and workers without a (permanent) employment contract had a reduced odds of sustainable RTW in the long run. This might be explained by social-political factors, such as ageing of the workforce, the availability of jobs in the labor market and the increase of flexible employment relationships [51]. RTW programs and practices should take this larger social-political context into account. Therefore, research aiming to investigate facilitators and barriers for RTW of more vulnerable groups of sick-listed workers can be highly recommended.
